# Climate change: A driver of increasing vector-borne disease transmission in non-endemic areas

**DOI:** 10.1371/journal.pmed.1004382

**Published:** 2024-04-04

**Authors:** Shlomit Paz

**Affiliations:** School of Environmental Sciences, University of Haifa, Haifa, Israel

## Abstract

In this Perspective, Shlomit Paz discusses the link between climate change and transmission of vector-borne diseases in non-endemic areas.

*Climate change can affect human health in complex ways, both directly (e.g., exposure to extreme temperatures) and indirectly (e.g., changes in infectious disease ecology), compounded by a multiplicity of biological, ecological, and socioeconomic factors*.

The transmission of vector-borne diseases (VBDs) is highly complex and multifactorial, and is impacted by a multiplicity of biological, ecological, socioeconomic, demographic, and human-caused factors, including climate, migration, global trade, and travel among many others. Although climate is one of several drivers, it is recognised as a major environmental factor influencing the distribution of VBDs. Climate change exacerbates the risk and burden of both vectors and pathogens and allows their introduction and dispersion into new regions [1]. Disease vectors (predominantly mosquitoes and ticks) capable of transmitting VBDs rely on external sources of heat to maintain their temperature within functional limits. As such, climatic conditions are major determinants in the physiology, ecology, development, and behaviour of vectors and also influence biological processes in the life cycles of pathogens [1,2]. When temperatures rise, these biological processes may be accelerated. For example, during heat waves, high temperatures increase the biting rate of female mosquitoes. Since disease transmission to humans occurs during blood feeding, higher biting rates lead to higher disease incidence [2]. Although the interaction between climatic variables and VBD transmission is complex, often nonlinear, and variable among different vector/pathogen combinations, there is clear evidence supporting an association between climate change and VBD transmission [3].

Malaria and dengue remain of significant concern, with 249 million malaria cases in 2022 [4] and 740.4 dengue cases per 100,000 population globally in 2019 [5]. However, substantial declines have occurred in recent years which can be attributed to economic development and the success of public health interventions. Between 2000 and 2019, malaria case incidence declined globally from 81 to 57 per 1,000 population at risk and malaria deaths decreased by one-third. Increased use of dual-ingredient insecticide-treated bed nets, improved diagnostic testing, and expanded access to artemisinin-based combination therapies contributed to this decline [4]. These achievements demonstrate the ability to reduce infectious disease transmission and highlight the difficulty in conclusively attributing and quantifying the impact of climate change as one of many complex factors influencing VBD transmission.

Climate change is a contributory factor in the expansion of geographical distribution of VBDs, since warmer conditions facilitate the establishment of vectors in new regions. Currently, tropical species are spreading towards the poles, and species are established at higher elevations due to the rising temperatures. As a result, we now observe the spreading of disease vectors to new, including non-endemic, areas due to improved (warmer) habitat suitability [6]. Pathogens may be dispersed into non-endemic regions through travel, trade or migration, whereas autochthonous transmission (i.e., cases with no travel history 2 weeks before the disease) can occur in areas where the vector is established and climatic conditions are favourable for transmission [1]. For example, global warming increases climatic suitability for *Aedes albopictus*, one of the vector mosquitos of dengue, which is adaptable to urban, rural, and agricultural habitats, and can breed in natural and human-made containers. Since *A*. *albopictus* can survive at below-freezing temperatures, it has the capacity to colonise a broader latitudinal gradient [2]. In parts of Europe, rising temperatures make conditions more suitable for virus transmission by *A*. *albopictus* since, among other processes, the link between higher temperatures and lower extrinsic incubation periods (the time between infection and the onset of symptoms) increase the vectorial competence (vector’s ability to transmit a pathogen) [1]. Following heat waves in 2022, the number of autochthonous cases of dengue in France reached their highest ever recorded [7].

Other examples of VBD transmission to non-endemic areas are the spreading of West Nile virus in Europe following heat waves [1] and the geographic expansion in Canada of *I*. *scapularis*, the main vector of *Borrelia burgdorferi* (a cause of Lyme disease), following elevated temperatures [8]. With warming temperatures, an expansion of malaria has been observed in both elevational and latitudinal directions within Africa. From 1898 to 2016, malaria mosquito vectors (*Anopheles* spp.) ranges gained an average of 6.5 m of elevation per year, and the southern limits of their ranges moved poleward 4.7 km per year. These findings are consistent with expectations for climate-linked range shifts [6].

The examples above emphasise the pressing need for prevention, preparedness, and decisive action in response to climate-sensitive VBDs by health authorities. This should include various actions such as data collection and surveillance, epidemiological investigations of cases, vector/pest control policies and action plans, and increased monitoring in high-risk regions/periods [9]. Since vaccines and curative treatments are currently lacking for most VBDs, such as West Nile fever, Zika, and dengue (for which the current vaccine only protects individuals with past infection), vector control and prevention to minimise human contact with the vector are crucial for protecting individuals and populations [10]. Substantial progress has been made towards the prevention of malaria with 2 vaccines (R21/Matrix-M and RTS,S/AS01) now recommended by the World Health Organisation (WHO) for the prevention of malaria in children living in regions with *P*. *falciparum* malaria transmission, with the potential to save hundreds of thousands of lives [11].

Continued efforts to tackle the increased transmission of VBDs will require inter-sectoral collaboration between governments, nongovernmental organisations, and experts from diverse clinical and scientific disciplines, including immunology, entomology, clinical microbiology, and climate and health scientists [1]. Since VBDs can spread rapidly across national boundaries, collaboration between countries to control cross-border VBDs and share insights into challenges relating to disease transmission should be a priority for health agencies, even where diplomatic relations are strained or absent.

Public health campaigns must go further to improve awareness of infection risk, disease symptoms, and prevention strategies, such as the use of mosquito nets and the importance of eliminating small breeding sites for mosquito larvae and pupae in water. To help communities adapt to the increased risks of VBDs, it is important to build strong community-level systems based on collaboration between officials and stakeholders, involving community leaders and health care workers with local knowledge [12]. Special attention should be directed to vulnerable populations such those as in refugee camps, which are at very high risk of outbreaks due to high population density, poor sanitation, and inadequate access to health and social services [2].

According to climate models, future climate change is expected to render additional areas suitable for the survival of vector species, due to worsening warming trends [1,2] in parallel with degradation of other interconnected support systems such as water, soils, and ecosystems that also influence VBD ecology. Predictive modelling of the expected impacts of climate change on VBD transmission involving different climate and socioeconomic scenarios is critical for the development of improved early warning systems for outbreaks and to help decision-makers evaluate where or when infections will emerge or spread [1]. Without effective surveillance, intervention and cross-border collaboration, the changing climate will continue to contribute to the risk of morbidity and mortality from VBDs, even in non-endemic regions.
